# Tumeur stromale à localisation rétro-rectale: entité macroscopique et difficultés chirurgicales

**DOI:** 10.11604/pamj.2018.30.154.10801

**Published:** 2018-06-20

**Authors:** Driss Erguibi, Abdelilah El bakouri, Yassine Fahmi, Bouchaib Kadiri

**Affiliations:** 1 Service de Chirurgie Générale (Aile I), Faculté de Médecine et de Pharmacie Hassan II, Casablanca, Maroc

**Keywords:** Rectum, tumeur stromale, immunohistochimie, traitement, Rectum, tumor stromal, immunohistochemistry, treatment

## Abstract

Les tumeurs stromales gastro-intestinales sont des tumeurs mésenchymateuses peu fréquentes dont la localisation rectale est extrêmement rare, et posent en pré-opératoire un problème de diagnostic et de prise en charge thérapeutique. Nous rapportons le cas d’une tumeur stromale à localisation rétro-rectale afin de bien étayer les particularités cliniques, radiologiques et thérapeutiques de cette entité rare.

## Abstract (en)

Gastrointestinal stromal tumors are uncommon mesenchymal tumors that very rarely occur in the rectum. They pose preoperative diagnostic and therapeutic problems. We report the case of retrorectal stromal tumor in order to highlight the clinical, radiological and therapeutic features of this rare entity.

## Introduction

Les tumeurs stromales gastro-intestinales sont des tumeurs mésenchymateuses peu fréquentes. Elles siègent le plus souvent au niveau de l’estomac (50-70%) et de l’intestin grêle (20 - 30%). La localisation rectale est extrêmement rare; elle représente seulement 5% des cas et 0,1% de toutes les tumeurs rectales [1,2]. A travers cette observation ainsi qu’une brève revue de la littérature, on propose d’étudier les particularités anatomo-cliniques, radiologiques et les difficultés chirurgicales de cette entité rare.

## Patient et observation

**Figure 1:**
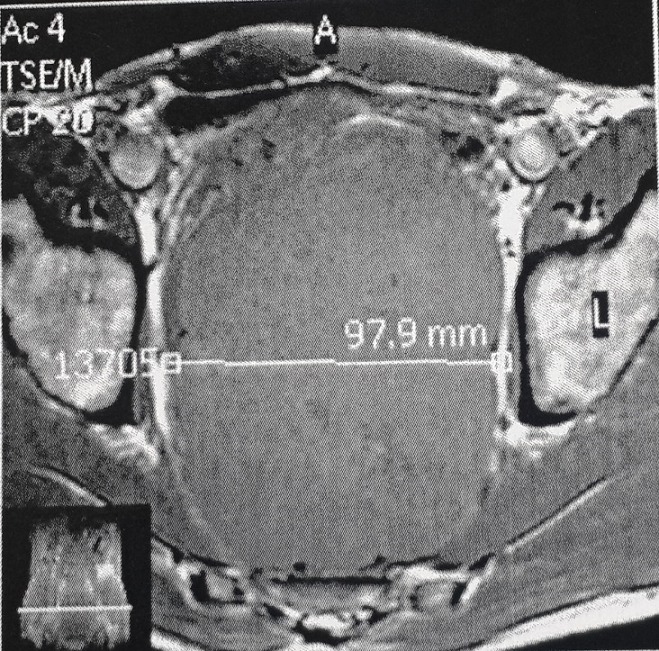
IRM pelvienne: volumineuse masse pelvienne basse, tissulaire, de 16 cm de grand axe, se rehaussant spontanément après injection de produit de contraste

**Figure 2:**
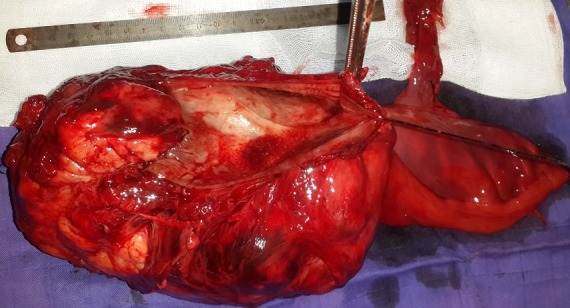
Pièce opératoire après résection

Un homme de 36 ans, pesant 63kg pour une taille de 1,70m, sans antécédent personnel particulier, avait présenté un syndrome rectal fait de ténesme et épreinte, sans hémorragie digestive extériorisée. Le toucher rectal percevait cependant une masse à la face antérieure du tiers inférieur du rectum, ferme et d’allure sous-muqueuse. L’examen clinique était normal, de même que les examens biologiques standards. La coloscopie complète ne mettait en évidence aucune autre anomalie en dehors d’un bombement dans la lumière rectale, sans occasionner de sténose, et d’allure plutôt sous-muqueuse qu’extrinsèque. Une IRM pelvienne a été réalisée montrant une volumineuse masse pelvienne basse, tissulaire, de 16 cm de grand axe, se rehaussant spontanément après injection de produit de contraste (Figure 1). Une résection de la tumeur avec anastomose colo-anale était réalisée malgré plusieurs difficultés chirurgicales (Figure 2). Le diagnostic de tumeur stromale a été retenu après étude anatomopathologique de la pièce d’exérèse chirurgicale, Sur le plan histologique, sous une muqueuse non ulcérée et une sous-muqueuse respectée, se développait, entre les fibres musculaires lisses, une tumeur à cellules fusiformes d’architecture fasciculée. Les mitoses étaient en nombre augmenté (mitoses pour 50 champs). Les cellules tumorales exprimaient fortement le CD 117 et le CD 34. Par endroits, la tumeur contenait des petites plages de nécrose dissociées par une infiltration hémorragique. La tumeur n’envahissait ni la sous-séreuse ni la séreuse. Compte tenu de la taille et de l’activité mitotique de la tumeur, le diagnostic de tumeur stromale du rectum à haut potentiel de malignité était donc retenu. Les suites postopératoires étaient simples autorisant la sortie au sixième jour ainsi le patient a été mis sous Imatinib en adjuvant avec rémission complète avec un recul de 2 ans.

## Discussion

Les tumeurs stromales gastro-intestinales (Gastro-Intestinal Stromal Tumors (GIST)) sont des tumeurs primitives digestives mésenchymateuses qui semblent se développer à partir des cellules interstitielles de Cajal des plexus myentériques, et expriment un récepteur transmembranaire KIT [3]. La survenue de GIST rectale est sporadique dans la grande majorité des cas, mais il existe quelques prédispositions familiales, telles que la neurofibromatose de type I et d’exceptionnelles formes familiales [4]. Les symptômes des GIST rectales sont très peu spécifiques, et ne diffèrent pas de ceux des autres tumeurs rectales. La rectoscopie repère facilement une tumeur stromale endophytique réalisant une formation arrondie bombant sous une muqueuse normale ou ulcérée. Lorsque la tumeur est à développement exophytique, la paroi rectale en regard peut apparaitre simplement rigidifiée posant un problème diagnostique comme dans notre observation [1,5]. L’échoendoscopie joue un rôle primordial en matière de GIST rectale, en effet elle permet de visualiser la tumeur qui se présente généralement sous forme d’une masse arrondie ou ovalaire, plus au moins hypoéchogène siégeant dans la quatrième couche (musculeuse) ou la troisième couche (sous-muqueuse) [6]. L’échoendoscopie a donc plusieurs intérêts dans l’étude préopératoire des GIST, elle permet une étude morphologique supérieure aux autres techniques d’imagerie en précisant sa taille, la tunique intra-pariétale dans laquelle est développée la lésion, pouvant ainsi orienter vers sa nature musculaire. Elle permet de préciser l’extension loco-régionale en recherchant une extension extra-rectale et les éventuels ganglions péri-rectaux. Elle contribue aussi à prédire le potentiel malin de la tumeur: une taille supérieure a 10 cm, la présence d’une nécrose centrale, l’envahissement des organes de voisinage et la présence des zones kystiques intratumorales [5,6]. Elle permet aussi de réaliser des biopsies par forage pour les tumeurs sous muqueuses avec un taux de positivité de 80 à 90% [6].

Le scanner abdominopelvien et surtout l’IRM permettent de bien étudier les GIST rectales à développement exophytique et de détecter un envahissement des organes de voisinage. Enfin, l’examen clé de confirrmation diagnostique reste toujours l’étude anatomopathologique avec un complément d’étude immunohistochimique. La tumeur doit exprimer le CD 117 en immunohistochimie (marqueur de la tyrosine kinase du récepteur KIT). Une caractéristique histologique importante est l’activité mitotique, conditionnant le potentiel malin des tumeurs [7]. Les GIST rectales nécessitent une prise en charge multidisciplinaire. La résection chirurgicale complète en monobloc de la tumeur (résection R0) est le seul traitement curatif des tumeurs stromales digestives. Il est essentiel d’éviter une perforation peropératoire qui entraine une dissémination péritonéale et une survie similaire à celle des patients ayant eu une exérèse incomplète dans certaines études. En outre, les énucléations simples sont grevées d’un risque de récidive significativement plus élevé que les résections segmentaires. En cas d’exérèse incomplète (R2) ou d’exérèse de nodules métastatiques péritonéaux associés, le pronostic spontané est mauvais. Le cas des résections R1 reste l’objet de discussions, car il n’a pas été formellement démontré qu’une résection R1 était associée à un moins bon pronostic [8]. Le curage ganglionnaire n’est pas systématique, car les métastases ganglionnaires sont rares et le risque de récidive ganglionnaire est limité [1]. En postopératoire, un traitement à base d’Imatinib est indiqué pour les formes a risque élevé ou intermédiaire de récidive et discute en cas de résection incomplète. Le pronostic des GIST rectales demeure réservé, la survie à cinq ans est entre 22 et 66% respectivement pour les tumeurs de haut grade et de bas grade de malignité [1].

## Conclusion

Les GIST rectales sont des tumeurs très peu fréquentes. Le diagnostic repose sur l’histologie et l’immunohistochimie. L’échoendoscopie a un intérêt majeur à la fois diagnostique, pronostique et de surveillance. La chirurgie est le traitement de choix pour les formes localisées pour les tumeurs du haut et moyen rectum. La chimiothérapie adjuvante à base d’Imatinib a améliorée la survie des patients avec des GIST rectales localement évoluées, métastatiques et des formes a haut risque de récidive. Le traitement néoadjuvant à l’Imatinib reste controverse. D’autres études sont nécessaires pour mieux clarifier la stratégie thérapeutique la plus efficace pour les patients atteints de GIST rectales.

## Conflits d’intérêts

Les auteurs ne déclarent aucun conflit d'intérêts.
